# Editorial: Polymers for high electric field applications

**DOI:** 10.3389/fchem.2023.1157986

**Published:** 2023-03-10

**Authors:** Jinghui Gao, Junwei Zha, Yongbin Liu, Davide Fabiani, George Chen

**Affiliations:** ^1^ State Key Laboratory of Electrical Insulation and Power Equipment, Xian Jiaotong University, Xian, China; ^2^ Department of Polymer Science & Engineering, University of Science and Technology Beijing, Beijing, China; ^3^ Department of Electrical Electronic and Information Engineering, University of Bologna, Bologna, Italy; ^4^ Electronics and Computer Science, University of Southampton, Southampton, United Kingdom

**Keywords:** polymer, energy storage, insulation, interface, high electric field

Polymers have been found to have wide applications in power equipment, due to their excellent electric insulation properties, energy storage performance, and mechanical behaviors. The continuous development of electric systems towards higher voltages, larger capacity, miniaturization, and a stringent environment has posed an increasing challenge for the investigation of polymeric materials. Attention has been directed towards polymeric dielectric properties, charge transportation/space charge, electric breakdown, interfacial phenomenon, and treeing, etc., to increase electric performances. These properties rely on the morphological and molecular structure of the polymer, particularly on its crystalline and amorphous phase and long-period and interfacial structures. The authors of this Research Topic have reported the in-depth investigations being conducted in this field.

For power cable insulation polymers, the working voltages for the manufactured cable go up to 500 kV for HVAC and 640 kV for HVDC applications, and the insulation thickness is enlarged, leading to difficulty in the removal of the byproducts caused by the crosslinking of polyethene (PE). In the case of the HVDC cable, the residual crosslinking byproducts may significantly influence the electric field distribution by affecting the conductivity of crosslinked polyethene (XLPE). F. Li et al. have reported a phase field model to quantitatively calculate the migration of crosslinking byproducts during degassing, considering the Fickian diffusion and uphill diffusion. The electric field distortion caused by the non-uniformity of byproduct distribution in cable insulation has been further evaluated. Another aspect is that the crosslinking network structure of XLPE makes the extruded cable insulation unrecyclable, which may cause pollution for the cables that are out of service (Li et al., 2022a). L. Li et al. have designed a blending material system consisting of linear low-density polyethylene (LLDPE) and high-density polyethylene (HDPE) forming a eutectic structure, which can be considered as a potential replacement of XLPE in the future. The long-term reliability of such a material system has been evaluated through investigations of its electric treeing and thermal aging phenomenon. The results show the LLDPE–HDPE blending material exhibits better anti-aging performance because of its large crystallinity, with a uniform and fine spherulite structure compared to XLPE, demonstrating that blending materials are promising environmentally friendly candidates for XLPE (Li et al., 2022b).

For energy storage dielectric polymers, the increasing demand for device miniaturization is resulting in the development of high-energy-density dielectric polymers. The energy density can reach around 3000J/L for the commercialized polypropylene (PP)-based high-voltage pulse capacitors and is expected to show a higher value for novel material systems. Most attempts are focused on how to enhance the dielectric permittivity and breakdown strength. M. Jin et al. have designed a lamination structure of PP/MgO and PP/BaTO_3_ nanocomposite dielectrics, which enhances the dielectric permittivity and breakdown strength simultaneously, and the energy density can reach 3.1J/cm^3^ (Ji et al., 2022). G. Meng et al. have laminated ultrathin hexagonal boron nitride (h-BN) on the surfaces of polyvinylidene fluoride (PVDF), forming a sandwich structure, and the energy density is 19.256J/cm^3^ (Meng et al., 2022). Another aspect is that high-permittivity polymers, such as PVDF, suffer from relatively high loss and temperature instability. Y. Liu et al. have proposed a method to develop a blending material for the *in situ* polymerization of methyl methacrylate (MMA) monomers in PVDF, and a high energy density of 8J/cm^3^ has been achieved at a temperature of 90°C (Liu et al., 2022).

Epoxy resins have also been found to have wide applications in the insulation of power equipment such as insulators, bushings, and wide bandgap semiconductor power devices. The space charge and the associated trap characteristic are important factors for the application of epoxy resins. H. Zhang et al. have reported the evolution of space charge behaviors for epoxy resins with a 24 h electric field load, and the results show the space charge accumulation cannot meet a stable state within 24 h at a temperature below 60°C, which suggests that a long-term space charge evaluation is necessary for the insulation of HVDC or UHVDC power equipment (Zhang et al., 2022). C. Wang et al. have studied the trap characteristics for specific liquid rubber toughened epoxy resins through the thermally stimulated depolarization current method, finding that the trap energy level increases with an increasing rubber concentration, which may be considered in the application in such an epoxy resin system (Wang et al., 2022). C. Chen et al. have investigated space charge accumulation for the SiC–epoxy resin interface, which may affect the insulation reliability of packaging materials (Chen et al., 2022).

In general, the development of polymers in high electric field applications stems from the engineering requirement for high voltages and electric insulation, and the deep scientific understandings of the materials and the associated high-performance polymers still remain an open question.
